# Domestic Violence and Mental Health

**DOI:** 10.1192/pb.bp.113.045385

**Published:** 2014-08

**Authors:** Floriana Coccia

The aim of this book is to offer practical guidance on how to identify domestic violence and respond to patient disclosures in a clinical setting. It is aimed at all mental health professionals, although general practitioners are likely to find it a valuable resource.

The book covers the bi-directional association between mental health and domestic violence and its prevalence. The following chapters offer multiple suggestions on aiding disclosures of domestic violence, as well as when and how to ask. They give practical advice on creating the right environment to allow disclosure and suggestions on how to broach the subject and then explore it further. The authors advise on how to respond to disclosures and provide examples of good practice as well as discussing undertaking assessments in difficult situations. The final chapters contain guidance on intervening for patients who have made disclosures and cover medico-legal definitions related to domestic violence.

There is limited evidence available for the authors to make use of, but what is available is well referenced and where evidence is lacking, good practice guidance is based on the authors’ own considerable expertise. The appendices have lists of contacts and organisations that offer support to those experiencing domestic violence and other resources for professionals to make use of.

I found the chapters on prevalence and the association between domestic violence and mental health a little overwhelming as the literature is very thoroughly documented. The following chapters are very helpful and address the common errors that well-meaning professionals might make, highlighting the barriers to disclosures of domestic violence. Guidance is offered on how and why to ask about domestic violence, with example questions and statements to improve professionals’ skills. The chapters on responding and intervening offer suggestions on what to avoid doing or saying as well as where to gain support, safety and advocacy for patients. The risks associated with victims attempting to leave their partners are also addressed. The final chapter is slightly more specialised and gives advice for those attending court, and will appeal to a smaller, more specialised audience.

Overall, the book is well written, thoroughly researched and has already changed my practice. Every mental health professional should have the skills to manage domestic violence and this book will be a valuable addition to anyone’s personal library.

